# The Early Movers Clinician's Guide: Co‐Designing a Resource for the 24‐Hour Movement Guidelines in Paediatric Occupational Therapy

**DOI:** 10.1111/cch.70275

**Published:** 2026-04-14

**Authors:** Leah G. Taylor, Sophie M. Phillips, Denis Tzvetkov, Liliana Alvarez, Patricia Tucker

**Affiliations:** ^1^ Health and Rehabilitation Sciences Western University London Canada; ^2^ School of Occupational Therapy Western University London Canada; ^3^ School of Kinesiology Western University London Canada; ^4^ Children's Health Research Institute, Lawson Health Research Institute London Canada

**Keywords:** clinical tool, codesign, disability*, knowledge translation, movement behaviourpreschool*

## Abstract

**Background:**

Balancing physical activity, sedentary time and sleep is important for the well‐being of preschoolers (3–4 years) with disabilities. Occupational Therapists (OTs) are important disseminators of the Canadian 24‐Hour Movement Guidelines for the Early Years but require a resource for clinical implementation. This study aimed to co‐design and assess the content validity of such a resource.

**Methods:**

Using the Framework for Co‐design of Clinical Tools, five workshops were conducted with an advisory council of OTs (*n* = 9) and caregivers of children with disabilities (*n* = 5) to co‐design the resource. Researchers (*n* = 6) consulted on the scientific integrity of the resource. Thematic analysis was used to summarize workshop data for the co‐design process and examine content validity.

**Results:**

The *Early Movers Clinician's Guide*, an open access resource to support OTs in using the guidelines was created. Themes reflected within the co‐design process included the importance of a person‐centred approach, meaningful measurement and context‐driven information sharing. Participants reported positive perceptions of the resource's content validity, supporting its potential for clinical application.

**Conclusions:**

The *Early Movers Clinician's Guide* was co‐designed to support OTs in translating the guidelines into practice with preschoolers with disabilities. Future research will evaluate broader implementation.

## Summary

1. Occupational therapists (OTs) are well‐suited to implement the 24‐h movement guidelines among their preschool‐aged clients (3–4 years) with disabilities.
2. A person‐centred, flexible and strength‐based approach is essential for translating movement guideline recommendations into meaningful, family‐centred occupational therapy practice.
3. Co‐designing a practice resource with clinicians, caregivers and researchers improved the clinical applicability of the guidelines for OTs who work with preschoolers with disabilities.
4. The *Early Movers Clinician's Guide* demonstrates potential to improve guideline implementation in paediatric occupational therapy practice.

## 1 Introduction

Engaging in physical activity, sedentary time and sleep habits has been positively associated with young children's short‐ and long‐term health and development (Kuzik et al. 2017). The Canadian 24‐Hour Movement Guidelines for the Early Years (0–4 years) were developed to provide daily recommendations for an optimal balance of these three behaviours (Tremblay et al. 2017). While the movement guidelines are supportive of the well‐being for all children, research suggests that children with disabilities generally have lower levels of physical activity, higher rates of sedentary and screen time and shortened sleep (Reynolds et al. 2019; Taylor et al. 2023; Vanderloo et al. 2025). As such, efforts are needed to support these behaviours in this population to ensure all young children receive the benefits of engaging in healthy movement.

Healthcare providers have been identified as key messengers of the guidelines (Faulkner et al. 2016). Occupational Therapists (OTs) are well‐suited to implement the guidelines with preschoolers (aged 3–4 years) with disabilities due to their focus on promoting participation in movement‐based play and sleep and their family‐centred practice (Taylor, Bourke, et al. 2025). Despite the role alignment, most OTs are not currently using the guidelines as they have reported a lack of training, tools and self‐efficacy to apply this resource in practice (Taylor, Bourke, et al. 2025; Taylor, Loh, et al. 2025). OTs have suggested a resource for paediatric OTs with a person‐centred approach would help support use of the guidelines in practice (Taylor, Bourke, et al. 2025; Taylor, Loh, et al. 2025).

For resources to be effectively implemented in practice, early and continued engagement with end users is crucial to ensure clinician priorities are addressed in the design process (Slattery et al. 2020). Creating a partnership among those with a vested interest (e.g., OTs, caregivers and researchers) is instrumental to the development of a resource to ensure user voices are heard (Vargas et al. 2022). By gaining a deeper understanding of the practice context of clinicians, and by employing an iterative co‐design process, user‐centred approaches can greatly improve the implementation and translation of evidence‐informed interventions (Dopp et al. 2019). Co‐design approaches in health research have been linked with improvements in both research processes and clinical practice outcomes, by reducing misalignment between fields (Slattery et al. 2020). Co‐design activities can also engage end users in the evaluation. Co‐designed evaluations ensure that resources developed will adequately address constructs of interest when implemented in practice (Mokkink et al. 2010). Evaluating the content validity of a resource is an important first step to supporting implementation of evidence‐informed interventions and future assessments of the reliability, validity and responsiveness of the resource (Terwee et al. 2018).

Given the potential of OTs to implement guidelines, the lack of uptake of the guidelines by clinicians, and the complementary addition of the guidelines to the current OT care, research is needed to investigate *how* to integrate them into practice. Therefore, the primary objective of this study was to work in partnership with OTs, caregivers and researchers to co‐design a resource for OTs working with preschoolers (aged 3–4 years) with disabilities to implement the Canadian 24‐Hour Movement Guidelines for the Early Years. The secondary objective was to explore initial participant perceptions of the content validity, usability and acceptability of the resource.

## 2 Methods

All study procedures were approved by Western University's Health Sciences Research Ethics Board (REB #125723) and informed consent was obtained from all participants. This study followed the Framework for Co‐Design of Clinical Tools (FRESCO), which facilitates a standardized 5‐step pragmatic and flexible process to create clinical practice resources (i.e., (1) predesign; (2) predesign, generative; (3) prototyping, generative; (4) prototyping, evaluative; and (5) evaluative) (Woodward et al. 2024). FRESCO Stage 1 (predesign) involved establishing a multidisciplinary advisory council. The authors (LT, SP, LA and PT) were the council's leadership team who were responsible for implementing all study procedures.

### 2.1 Participants

The study participants were comprised of two groups: the advisory council and the consultants. The advisory council consisted of OTs (*n* = 9) and caregivers of children with disabilities (*n =* 5). This group was responsible for coleading the development and design of the resource during four of five workshops. The advisory council was recruited based on their self‐identified expertise from practice and lived experience. OTs recruited were registered to practice in Canada and worked with children aged 3–4 years with disabilities. Caregivers recruited were the primary guardians of a child under the age of 8 with a disability who accessed occupational therapy services during the child's preschool years (ages 3–4; i.e., < 5 years postservice acquisition). Advisory council members were recruited via the Canadian Association of Occupational Therapists' website, social media and snowball and purposive sampling in professional and disability community networks. Purposive and snowball sampling are typical in co‐design research, given the need to draw on lived experience, establish commitment to the research project and build relationships with participants (Whitmore et al. 2024).

The consultants were field researchers from across Canada (*n* = 6), who were recruited via direct contact from the primary investigator (PT). Consultants were recruited from academic and non‐profit organizations because they were considered topic experts possessing critical knowledge in the areas of focus for this research (i.e., early‐childhood movement behaviours, disability, occupational therapy and knowledge translation). During two of the five workshops, the consultants offered support for resource creation (including crafting the language), which met the needs identified by the advisory council, while also ensuring the content maintained the integrity and accuracy of the original guidelines and its evidence base. Inclusion criteria for all participants are provided in Table 1. Lived experience of participants and representation from across Canada were prioritized versus completing a sample‐size calculation (Hennink and Kaiser 2022).

**TABLE 1:** Inclusion criteria by participant group.

Participant group		Inclusion criteria
**Advisory council**	**Occupational therapists**	Currently registered with a provincial occupational therapy regulatory body with the ability to practice in Canada Self‐determined experience working with paediatric patients (aged 3–4 years) with disabilities Able to speak, read and write in English Access to the internet.
	**Caregivers**	Self‐identified primary caregiver of a child under the age of 8 years with a disability (i.e., < 5 years postoccupational therapy experience) Self‐identified experience using occupational therapy for their child, during the child‘s preschool years (ages 3–4 years) Able to speak, read, and write in English Access to the internet
**Research consultants**		Able to speak, read, and write in English; Access to the internet.

### 2.2 Procedure

**FIGURE 1:**
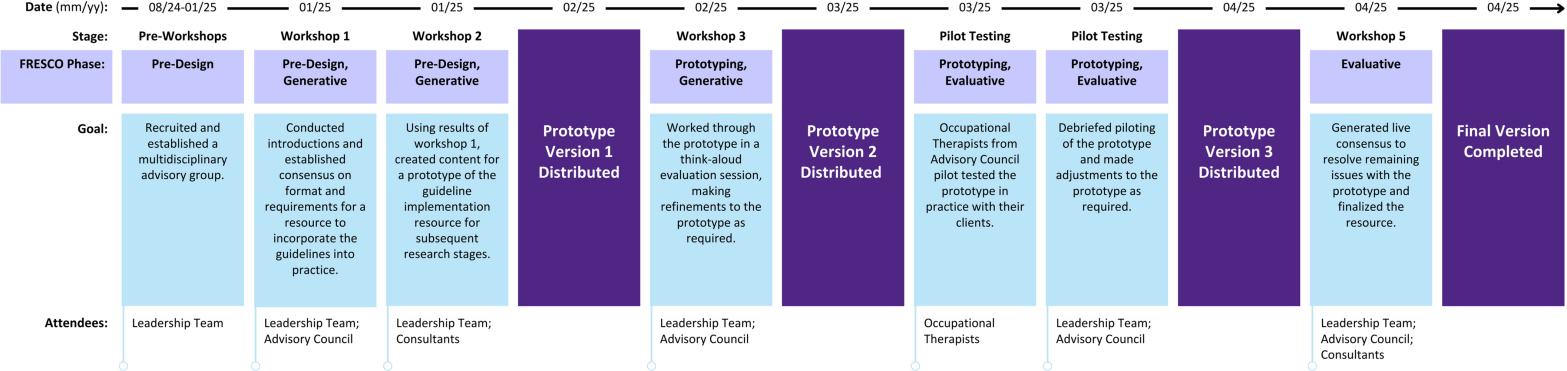
Co‐design progression, goals and timelines.

To address the first objective, five, 60‐min co‐design workshops were carried out using Microsoft Teams (see Figure 1—Study Timeline). The first and second authors facilitated the workshops by providing an agenda and prompting discussion among participants. Participants were encouraged to share any information they felt relevant with the group. Facilitators would ask follow‐up questions, make summarizing statements and keep the group on task during the conversations. Workshops were audio/video recorded and transcribed verbatim. Transcriptions were verified by one of the authors (DT). Between workshops, the primary author reviewed workshop transcripts and summarized all workshops in meeting minutes, which were used to create and update the co‐designed resource versions (i.e., prototype). Minutes and prototype iterations were distributed to the leadership team and advisory council for review between workshops. The advisory council was also invited to provide anonymous, spontaneous feedback between workshops in an open text box survey on Qualtrics.

FRESCO Stage 2 (*predesign* and *generative*) was achieved in Workshops 1 and 2. Workshop 1 entailed the advisory council and leadership team meeting to review the current guidelines. The group engaged in conversations on requirements for the content of a resource to incorporate the guidelines into occupational therapy for preschoolers with disabilities. In Workshop 2, the consultants met with the leadership team to discuss the advisory council's suggestions and drafted content for a prototype of the resource. Following Workshop 2, the first iteration of the prototype (Version 1) was developed by the leadership team.

FRESCO Stage 3 (*prototyping* and *generative*) occurred in Workshop 3 via think‐aloud evaluations of the prototype (Version 1). Participants worked through the prototype as a group while verbalizing their thoughts on the prototype as if they were experiencing it in a clinical setting, to understand how the resource could be used in practice (Li et al. 2012). To address Objective 2, the advisory council was asked to share their thoughts on the content validity of the prototype version (Version 1; see Table 2). Content validity was defined as the relevance, comprehensiveness and comprehensibility for the target population and context of use (Terwee et al. 2018).

**TABLE 2:** Definitions used to explore content validity.

Construct	Definition
Relevance	The resource's applicability to assess the construct of interest within the population of interest and context of use
Comprehensiveness	No key aspects of the construct of interest are missing
Comprehensibility	All resource items could be understood by clients as intended.

Based on the feedback from the think‐aloud session, the leadership team updated the prototype. The prototype (Version 2) was redistributed to the advisory council for FRESCO Stage 4 (*prototyping* and *evaluative*). To further explore Objective 2, for 3 weeks OTs tested the prototype (Version 2) in practice to further examine content validity, using clinical reasoning to implement the resource. OTs were instructed to use their clinical judgement to test the resource with clients aged 3–4 years with a disability. OTs then recorded the experience in a client‐unidentified, brief momentary reflection journal on Qualtrics immediately following the interaction to capture thoughts and experiences on the content validity, usability and acceptability when implementing the prototype (Version 2) in practice. Usability was defined as the degree to which an innovation may be used efficiently, effectively and satisfactorily to achieve goals in a specific context (International Organization for Standardization 2018). Acceptability was defined as the extent to which an innovation and its components are agreeable to the user (Proctor et al. 2011). Table 3 provides questions asked within the brief momentary reflection journal and the relevant construct being assessed.

**TABLE 3:** Brief momentary reflection journal questions to evaluate content validity, usability and acceptability.

Construct assessed	Question/prompt
Background on case	Provide the client deidentified case presentation at the time of prototype use. Briefly describe the child's current levels of (1) physical activity, (2) sedentary time and/or (3) sleep relevant to the conversation.
Resource use instance with client	What clinical interaction instance are you using the prototype with this child (e.g., first and second)?
Relevance	What are your thoughts on the **relevance** of the prototype when discussing the movement behaviours (e.g., physical activity, sedentary time and sleep) in the context of this client interaction? **Relevance** is defined as the resource's applicability to assess the construct of interest within the population of interest and context of use.
Comprehensiveness	What are your thoughts on the **comprehensiveness** of the prototype when discussing the movement behaviours (e.g., physical activity, sedentary time and sleep) in the context of this client interaction? **Comprehensiveness** is defined as no key aspects of the construct of interest are missing.
Comprehensibility	What are your thoughts on the **comprehensibility** of the prototype when discussing the movement behaviours (e.g., physical activity, sedentary time and sleep) in the context of this client interaction? **Comprehensibility** is defined as all resource items could be understood by clients as intended.
Usability	What are your thoughts on the prototype's ability to effectively integrate movement behaviour conversations into the context of this client interaction?
Efficiency	Roughly how long did it take you to administer the prototype in this client interaction? 0–4 min 5–9 min 10–14 min 15–19 min 20+ min
Acceptability	In this client interaction, what are your general thoughts of the prototype? Please consider: What went well when using the prototype? What did you like about the prototype? How do you think the prototype could be improved?
Additional reflections	Please provide any additional thoughts, feelings, or experiences not captured above.

In Workshop 4, the advisory council debriefed piloting the prototype (Version 2) in practice and reviewed the brief momentary reflection journals. Each member had the opportunity to share reflections on the prototype (Version 2) via a round table discussion. The reflection journals, anonymous feedback from Qualtrics and discussion in Workshop 4 were then used to inform updates to the prototype (Version 3). Open text survey responses on Qualtrics (*n* = 3) primarily related to ideas on useful resources to include in the prototype. These suggestions were added into the final prototype version for review by all participants. The resource was sent to the advisory council to reflect over the next 2 weeks.

In Workshop 5, advisory council members and consultants provided a final round of feedback and generated live consensus by voting on resolutions to remaining issues (FRESCO Stage 5 [*evaluative*]) (Humphrey‐Murto et al. 2017). Participants also completed a demographic survey on Qualtrics. The final prototype (Version 4), the *Early Movers Clinician's Guide*, was created following Workshop 5.

At the beginning of each workshop, the previous session was recapped and participants had the opportunity to bring up any new thoughts they felt had not been captured in the previous session. At the end of each session, facilitators also reserved time for participants to share any additional or previously undiscussed perspectives. In these instances, no new insights were raised. By the fifth workshop, no new topics or themes emerged, and consensus was reached on the final version of the prototype, indicating that sufficient depth and breadth of input had been obtained.

### 2.3 Data Analysis

The five workshops entailed an iterative process whereby the prototype was continuously evolving, informed by the conversations during each workshop. Transcripts from each of the workshops were summarized to highlight the key conversations informing prototype versions and ultimately the final resource. Thematic analysis, conducted in Microsoft Word, was then used to explore the co‐design process to create the resource (expanding on Objective 1) and the initial content validity, usability and acceptability of the resource (Objective 2) (Guest et al. 2014). Transcripts of Workshops 1 to 5 were independently, inductively coded by two of the authors (LT and DT), who met after each workshop was coded to discuss results and reach consensus before moving on to the next workshop to ensure intercoder reliability (Simons et al. 2008). A code book with definitions for each label was generated and updated by the authors at each meeting and carried forward (Creswell and Plano Clark 2017). If no existing code appropriately captured the data, authors could suggest that a new code be added. Confirmation bias was addressed as the second individual (DT) was not involved in the project directly and was only responsible for coding. A second round of coding was performed by the first author to gather similar concepts into themes. Quotations in each theme were reviewed by all authors to ensure trustworthiness (Anney 2014). Descriptive statistics were calculated (SPSS Version 29) to examine demographic characteristics of study participants (International Business Machines Corporation [IBM] 2022).

## 3 Results

Twenty participants from six Canadian provinces took part in this study to co‐design the *Early Movers Clinician's Guide*, an open access resource to support OTs in using the guidelines with preschoolers with disabilities. This included nine OTs, five caregivers and six research consultants. Table 4 provides complete participant demographic details. Briefly, the average years of OT practice was 18.6 years (SD = 8.2; Range = 7–34), with an average of 16.3 years working with paediatric populations (SD = 9.3; Range = 3–34). Caregivers reported their children's disabilities included attention‐deficit/hyperactivity disorder, autism, sensory processing disorder, traumatic brain injury, epilepsy and cortical visual impairment. Caregivers also reported their child is a wheelchair user (*n* = 1), and their child uses a gastrostomy tube for feeding (*n* = 1). All six consultants reported expertise in the 24‐h movement behaviours and knowledge translation, four in disability studies and three in occupational therapy. A summary of the workshop discussions is available in the Supporting Information.

**TABLE 4:** Participant demographics.

	Occupational therapists	Caregivers	Research consultants
	*n* = 9	*n* = 5	*n* = 6
**Gender** (Woman including trans woman; % of total participant group)	9 (100%)	3 (60%)	5 (83%)
**Racial background/ethnicity**			
White	8	2	5
Black	—	2	—
East Asian	1	—	—
Prefer not to answer	—	1	1
**Age** (Years; *M*, SD)	45.5 (8.6)	36.8 (3.4)	40.2 (5.0)
**Province of residence**			
British Columbia	1	1	—
Alberta	2	1	1
Saskatchewan	1	—	—
Ontario	4	3	2
Quebec	1	—	2
Nova Scotia	—	—	1
**Household situation**			
Double parent	—	4	—
Single parent	—	1	—
**Total annual household income (CAD)**			
$60 000–$79 000	—	1	—
$80 000–$99 000	—	2	—
Prefer not to answer	—	2	—
**Area of research expertise** (Select all that apply)			
24‐h movement behaviours	—	—	6
Knowledge translation	—	—	6
Disability	—	—	4
Occupational therapy	—	—	3

Abbreviations: — = question not asked of participant group; M = Mean; SD = standard deviation.

### 3.1 Co‐Design of the Early Movers Clinician's Guide

A thematic analysis of participant perspectives in the workshops was completed to illustrate the advisory council and consultants' priorities and values in the resource's development. Three themes recurring throughout the workshops described the advisory council and consultants' perspectives in creating the resource: (1) ensuring a person‐centred approach; (2) measurement through meaning; and (3) information sharing via connection and context. The way the resource generation reflected each theme is noted within each section.

#### 3.1.1 Ensuring a Person‐Centred Approach

When considering using the guidelines in practice, the advisory council had concerns about the applicability of the current format. They felt the prescriptive, one‐size‐fits‐all messaging of the guidelines did not capture the diverse needs of children with disabilities and their families. For example, an OT mentioned: ‘It's not quite as simple as just put some toys in front of them and they're gonna play with those toys … For some of the kids, they need the iPad at this point … to remain regulated’. Participants agreed with concerns surrounding limiting screen time, indicating the role of screens as a safe space, interactive and engaging tool for occupational participation and for supporting development. The advisory council felt the messaging did not consider diverse movement patterns for children with disabilities, with a caregiver saying, ‘Because what works for [child] A wouldn't necessarily work for [child] B, their disabilities are also an influencing factor’. Participants also had concerns with the ableist language surrounding limiting sitting and avoiding the use of restraints. An OT indicated, ‘Sitting and restrained is how [some children are] able to participate, and if you remove those supports, they're no longer able to function to the best of their ability … A seated posture and a supported posture are their best way of participating’.

When presented with these concerns regarding language in the guidelines, a consultant spoke to the origin of the guidelines: ‘It's not really made for this group … We've had a hard time on how do we get around that to still get the main message across that movement is important for all children including those with disabilities’. The consultants saw this as an issue of trying to assimilate the guidelines to individuals who were not considered in their creation, and where a lack of representation exists in the literature. A consultant noted: ‘I think that challenging the notions we have of what movement should look like … I feel like in this resource, it is redefining what movement is… Or different ways of moving when we think about young children with disabilities … Maybe currently the way it's defined it's not achievable for some children’.

All participants agreed person‐centred tools to explore and implement the guidelines with each client were the way forward. As stated by an OT: ‘Build some more flexibility into the guidelines saying everyone's going to be different … So work within what you have and just to start small’. Incorporating flexibility into the guidelines would allow OTs to tailor the messaging to the unique experience of each child and make it more complementary to their goals and interests. To make the resource more person‐centred and directed towards occupation, tools were created to facilitate messaging from strength‐based approaches. A continuum to explore energy expenditure was also used to describe how movement could be considered for each person.

#### 3.1.2 Measurement Through Meaning

The advisory council felt time‐based indicators were not appropriate to integrate the movement behaviours into practice. OTs on the advisory council noted the science behind numbers was helpful, but when communicating time‐based targets to caregivers, it could lead to feelings of judgement. An OT expressed: ‘When you put numbers in these kinds of categories it can be daunting for families to feel like that's the gold standard and then feeling guilty that they're not meeting those goals’. OTs and caregivers felt movement conversations with families of children with disabilities should be based around their own goals. For example, an OT said: ‘Sitting still is quite an accomplishment for many of the families I work with’. The advisory council was looking for flexible strategies for discussing activities, which aligned with the goals of the family and were supportive of overall well‐being. This was expressed by a caregiver: ‘In a household with a child with needs, no day is ever the same as the one before. If it had some language around flexibility instead of a determined amount of time it would help with the pressures of the document’.

To address these concerns, the consultants aimed to refocus the messaging to patterns of health‐supporting behaviours over time. A consultant suggested: ‘Those specific numbers don't really matter because at the end of the day, you want to prioritize sleep, increase movement in your day … but that 180 minutes, there is nothing magical about that’. The consultants suggested focusing on quality, rather than the quantity of movement behaviours. The consultants felt the Quality Participation Framework (Evans et al. 2018) aligned well with the advisory council's call for a way to assess meaningful activity. A consultant described this concept: ‘Within that framework it looks at six different elements of experiences … It exists, and there's been research done in sport, exercise and even in active play for kids with disabilities’.

Building on the person‐centred tools of the resource, the advisory council and consultants wanted to craft a resource that would integrate the scientific evidence of the movement behaviours, while providing strategies to integrate them meaningfully for families. A consultant explained: ‘So you have these recommendations but then the point of the resource is breaking them down. Like what does this mean and ensuring that idea that the flexibility and the ability to adapt them to the particular person that they're working with’. Strategies to craft this messaging included using the Quality Participation Framework and taking a harm‐reduction approach to support movement participation, which balances quality with quantity. This involved resources to gauge readiness for discussing the movement behaviours and worksheets to tailor interventions to the family's context.

#### 3.1.3 Information Sharing via Connection and Context

How OTs will engage in knowledge translation of the guidelines in their practice was an important consideration for creating a resource with relevance across the nation, but that could also be implemented locally. An OT explained: ‘Integrating different resources that I can kind of direct families to. The places in their areas that could be helpful would be interesting, I think’. Specifically, the advisory council felt information needed to be shared in a way that was useful for families, easy to access and attended to differences in client contexts. Resources that were included needed to account for programme availability, geography and seasonality. At a person level, the information being shared also needed to consider the client readiness to receive the information. This meant the OTs would need to use clinical judgement to ensure the right volume of content would be shared at the right time. A caregiver shared: ‘Considering what one of those categories was like plaguing me with, I appreciate that the OTs would be there to kind of gauge what are you ready to hear right now and what would be helpful, and how can I kind of bring this to you as you need it’. The advisory council felt that the goal should be breaking up information surrounding each behaviour to provide only the information that the family was ready for.

The feedback from the advisory council led consultants to consider the specific messaging of the guidelines for this audience. A consultant summed up the goal of knowledge translation for this resource: ‘People don't want numbers, they want tools’. To do this, the consultants felt creating two segments of content in the resource to provide researcher to clinician knowledge sharing and subsequent clinician to caregiver knowledge sharing was the way forward. A consultant said: ‘Just to start the discussion, I think it seems it would be necessary for one product for the clinicians with more details and a simpler version for them [the OTs] to give out to families’.

To address the knowledge translation goals, the resource was created in two parts. Part 1 is a clinician facing resource which provides education and background on the guidelines. Part 2 provides tools for engaging with caregivers. OTs are encouraged to take what they need from the resource to tailor the messaging to meet the needs of their clients. To create a resource that was easy to access and useful for families from across Canada, national and international information is provided with check boxes for OTs to draw attention to relevant information only. QR codes and live links were integrated to facilitate interaction with the included resource and space was provided, so OTs could fill in gaps with local programming.

### 3.2 Content Validity, Usability and Acceptability

#### 3.2.1 Content Validity

OTs felt positively about the content of the final version of the resource and how it would support them when working with preschoolers with disabilities. The advisory council and consultants aimed for the resource to frame movement in nuanced and strength‐based ways. For example, advisory council members felt that using an energy expenditure continuum to describe the movement behaviours was a person‐centred and strength‐based approach for use in practice. An OT indicated: ‘What might be appropriate for one child, and another may be very different as you move along that spectrum … That is really a powerful graphic and message’. The advisory council also felt that the resource was comprehensive in the use of links and QR codes to external resources, check boxes and space allocated to make the messaging relevant to a local setting. For example, an OT stated: ‘We got to circle things and add things, just to make it child specific’. OTs on the advisory council felt that using the resource in a session would take 15 to 20 min to talk generally. They suggested that picking one movement behaviour to focus on at a time was an appropriate way to maintain comprehensibility and prevent information overload.

#### 3.2.2 Useability

Six OTs were able to pilot test the prototype (Version 2) in practice, each with one client, between Workshops 3 and 4. Most (83%) reported it took 15–19 min to use the resource in practice with their client, and one OT reported it took them 5–9 min. The OTs felt positive about the resource's usability. For example, one OT stated: ‘The visuals are super helpful, and it [the resource] gives us the tools to be able to bring the information into session’. The tools available in the appendix were also linked to the positive feelings. When testing the resource, the OTs were able to facilitate conversations with families around their child's goals for movement and then tailor their interventions to address the family's needs. An OT noted: ‘We really focused more on the sleep because that's where he has more difficulty and I thought the resource was super helpful and concrete, like it gave concrete strategies for them to be able to try out’. Another OT focused on sedentary and screen time and used the resources to guide a conversation around harm‐reduction with their client: ‘They were like, I have this idea about screens always being this negative thing that we do … And I think it was helpful for parents to feel comfortable about certain things they did, and a conversation was able to be built around how we can support more benefits with certain aspects versus others’. Overall, the OTs felt the resource helped guide conversations and interventions on the movement behaviours in the context of their client's priorities.

#### 3.2.3 Acceptability

The advisory council felt positively towards the agreeableness of the final version of the resource for use in practice. A caregiver reported: ‘My OT would most likely give this to me as a resource. I enjoy them [the caregiver tools], they are easy to follow’. OTs appreciated the autonomy to use their experience coupled with the information provided to facilitate the conversations with families. An OT indicated: ‘I like the little blurb that the tool kit should be used in tandem with their clinical judgement…You're not saying this is a recipe that we're handing out or this is a prescriptive thing that we need to do … and instead starting discussions’. The advisory council conveyed that they felt the final product would be helpful for families.

## 4 Discussion

This study entailed co‐designing and developing the *Early Movers Clinician's Guide,* an open access resource to support OTs in using the Canadian 24‐Hour Movement Guidelines for the Early Years with preschoolers with disabilities. This study also captures the perspectives of OTs and caregivers on the initial applicability and usability of the resource. The *Early Mover's Clinician's Guide* offers OTs information and resources to discuss the movement behaviours with caregivers of preschoolers with disabilities. It also provides guidance that can be personalized to individual children and focuses on sensitive, timely and strength‐based ways to support integration of the guidelines in children's contexts. Drawing on the clinical judgement of OTs to integrate meaningful recommendations for each child, this work seeks to provide OTs with a resource to act as a dissemination avenue to support uptake of the health‐supporting guidelines (Faulkner et al. 2016). It also aims to be responsive to the concerns on guideline relevance for individuals with disabilities (discussed below). The perceptions of initial content validity from participants in the co‐design process indicate that the *Early Movers Clinician's Guide* can support contextually relevant conversations on movement behaviours through the research‐based recommendations and the resources provided, though future evaluation of the resource is required.

Research surrounding the guideline applicability for children with disabilities is debated in the literature. Some researchers express concerns surrounding the evidence to set population‐based benchmarks, which excluded children with disabilities, issues of measurement, ableist messaging and a lack of acknowledgement of diverse disability experiences and defining different intensities of movement accordingly (Arbour‐Nicitopoulos et al. 2022; Arbour‐Nicitopoulos et al. 2025; Martin Ginis et al. 2021; Smith et al. 2021). More work is needed before guidelines for children with disabilities can be created; however, when matched to a child's abilities, the movement behaviours are relevant and important for all children's development (Tremblay et al. 2017). Therefore, application of the guidelines can be used by families of children with disabilities in consultation with a healthcare professional to support their children's well‐being. The *Early Mover's Clinician's Guide* seeks to support this knowledge translation process for OTs working with preschoolers with disabilities.

Current lags in the translation of research evidence into healthcare practice are a global knowledge exchange issue to which the guidelines are not immune (Riazi et al. 2017). Collaboratively engaging with end users through opportunities to share their expertise, knowledge and skills can result in enhancing the relevance and the uptake of the research (Hoekstra et al. 2020). Using a co‐design approach allowed for the creation of a specific knowledge translation resource tailored to the needs of OTs working with preschoolers with disabilities. This approach also aimed to create an opportunity for meaningful engagement of members of the disability community to improve real‐world relevance of this work (Gainforth et al. 2021). Finally, this work sought to address concerns surrounding limits of training and understanding to disseminate the guidelines raised by OTs in previous studies (Taylor, Bourke, et al. 2025; Taylor, Loh, et al. 2025).

This study complements the work done by Verschuren et al. who employed coproduction methods to generate resources for healthcare providers counselling on the movement behaviours for children with cerebral palsy (Verschuren et al. 2021). Morgan et al. also created a resource for Canadian primary care providers to discuss the guidelines with their adult patients (Morgan et al. 2023). This resource has since been adapted for physiotherapists in Canada (Morgan et al. 2025). Although coproduction methods may have been better suited in this past resource development, a co‐design approach allowed for users to colead the design, implementation and evaluation to the prespecified need for resource development to address guideline integration in occupational therapy (Taylor, Bourke, et al. 2025; Taylor, Loh, et al. 2025). Drawing on the FRESCO process, we were able to engage in a systematic, user‐centred and transparent co‐design approach to facilitate pragmatic, flexible and inclusive practices to designing this resource (Woodward et al. 2024).

This study used a systematic and engaged co‐design process to develop the first resource for use of the guidelines in occupational therapy, with participation from clinicians, caregivers and researchers. Previous research provided recommendations for the development of such a resource, stating the resource should (1) be clinician and client facing; (2) facilitate individual guideline tailoring; (3) encourage meaningful movement; (4) support knowledge translation for families; and (5) be easy to administer (Taylor, Loh, et al. 2025). Perceptions of the advisory council indicated the *Early Movers Clinician's Guide* met these recommendations. However, there are limitations which must be acknowledged. In using workshops to co‐design the resource, social desirability bias could have affected the participant feedback. To mitigate this issue, a venue for anonymous feedback was provided to participants outside of group settings (e.g., open text box survey on Qualtrics). Moreover, while efforts were made to recruit participants from different parts of the country and with diverse experiences as clinicians and caregivers of children with a disability, the sample still largely identified as white women who resided in 6/13 provinces or territories in Canada. This means that diverse perspectives (including gender, ethnicity, geography and junior practitioners) may have been missed, which limits transferability of the results.

## 5 Conclusions

The Early Movers Clinician's Guide* represents a novel, co‐designed resource that supports OTs in integrating the guidelines in practice with preschoolers with disabilities. This work is an important step towards supporting movement behaviours for children with disabilities, and in recognizing the role that OTs can play in the field of exercise science. This resource offers a promising step towards enhancing guideline‐informed and equity‐focused care for preschoolers with disabilities. While the creation of this resource is a first step, additional research is needed to evaluate if this resource supports engagement among preschoolers with disabilities in movement behaviours in occupational therapy and examine the impact of this resource on clinicians' perceived levels of training, access to tools and self‐efficacy to apply the guidelines in practice.

*To view the open access *Early Movers Clinician's Guide*, please visit our website: https://www.childpalab.ca/currentprojects‐otstudy.

## Author Contributions

**Leah G. Taylor:** conceptualization, data curation, investigation, analysis, project administration, visualization, writing – original draft. **Sophie M. Phillips:** conceptualization, investigation, analysis, visualization, validation, writing – original draft. **Denis Tzvetkov:** data curation, analysis, validation, writing – review and editing. **Liliana Alvarez:** conceptualization, supervision, validation, writing – review and editing. **Patricia Tucker:** conceptualization, investigation, resources, supervision, validation, visualization, writing – original draft, funding acquisition.

## Funding

L.T.'s work is supported by a Canadian Institutes of Health Research—Canada Graduate Scholarship (Doctoral) and an Ontario Graduate Scholarship. S.P. is supported by the Canadian Institutes of Health Research—Postdoctoral Fellowship (FRN: 200967), Children's Health Research Institute, and Mitacs. Funding from Western University and the North American Society for Paediatric Exercise Medicine supported participant compensation and knowledge translation of this work. The Public Health Agency of Canada financially supported translation of the *Early Movers Clinician's Guide* into French. The Canadian Disability Participation Project financially supported document accessibility services to ensure usability by adaptive technology and compliance with global accessibility standards.

## Ethics Statement

All study procedures were approved by Western University's Health Sciences Research Ethics Board (REB #125723), and informed consent was obtained from all participants.

## Conflicts of Interest

The authors declare no conflicts of interest.

## Supporting information




**Data S1:** Summary of workshop discussions.

## Data Availability

Data supporting this study's findings are available upon reasonable request to the corresponding author.
