# Supplementary material for: The Early Movers Clinician's Guide: Co‐Designing a Resource for the 24‐Hour Movement Guidelines in Paediatric Occupational Therapy
Source: Child Care Health Dev. 2026 Apr 14;52(3):e70275. doi: 10.1111/cch.70275 (PMC13078964; doi:10.1111/cch.70275)
Supplement: Supplementary file 1 — Data S1: Summary of workshop discussions. [file CCH-52-e70275-s001.docx]

**Supporting Information: Summary of Workshop Discussions**

1. W**orkshop 1 (OTs, Caregivers, Leadership Team): P**articipants discussed the need for an implementation resource with inclusive and flexible language, avoiding rigid numerical targets, and acknowledging the diverse realities of families of children with disabilities. The advisory council wanted to reframe the recommendations to reflect functional use, allow tailoring to different ability levels, and support connection with community resources.
2. Workshop 2 (Researchers, Leadership Team): The consultants prioritized inclusive language tailored to children with disabilities, avoiding rigidity of the original guidelines to reframe messaging of the recommendations. Consultants felt promoting movement in a way which prioritized quality over quantity was important to meet the needs of the advisory council. Consultants recommended two resources, one clinician-facing and one caregiver-facing, as the most effective way to communicate the guideline messaging.
3. Workshop 3 (**OTs, Caregivers, Leadership Team)**: The advisory council provided feedback on the prototype (version 1). The main feedback included a need to ensure consistent, plain, and strength-based language throughout, and inclusive messaging and visual aids representing children with various abilities. Participants wanted more tools built into the resource to support clinicians and families in meaningful application of the guidelines.
4. Workshop 4 (**OTs, Caregivers, Leadership Team)**: After OTs pilot tested the prototype (version 2) in practice, the advisory council reconvened and were in favour of the variety of strengths-based tools, highlighting the usefulness in guiding individualized, family-centered discussions about movement behaviours. They emphasized the need to break the content into smaller, more manageable pieces, to better support tailoring strategies to families’ readiness and diverse needs (e.g., further dividing physical activity, sedentary time, and sleep sections in the parent resource, using checkboxes, and QR codes, etc.).
5. **Workshop 5 (OTs, Caregivers,** Researchers, **Leadership Team):** The advisory council and consultants made minor refinements to the prototype (version 3) such as clarifying terminology, ensuring inclusive language, and enhancing sensory-related content, which resulted in the development of the final document (version 4). They discussed knowledge translation strategies, emphasizing the importance of diverse dissemination channels (e.g., webinars, conferences, videos), and ensuring accessibility through features like text-to-speech and translation into French.
